# Dr. Harlan Retires

**Published:** 1894-02

**Authors:** 


### DR. HARLAN RETIRES.

THE January number of the *Dental Review*, Chicago, announces the retirement of Dr. Harlan as editor of that journal.

This is a serious loss to our contemporary, as it has been largely through his ability and untiring energy that has made this one of the best of the trade journals.

Dr. C. N. Johnson has been placed in the position vacated by Dr. Harlan. He has been connected for some time with the *Review* as assistant editor. Drs. T. L. Gilmer and George J. Dennis will aid as associate editors.

